# Vicarious representation: A new theory of social cognition

**DOI:** 10.1016/j.cognition.2020.104451

**Published:** 2020-12

**Authors:** Bence Nanay

**Affiliations:** Centre for Philosophical Psychology, University of Antwerp, D 413, Grote Kauwenberg 18, 2000 Antwerp, Belgium; Peterhouse, University of Cambridge, Cambridge CB2 1RD, UK

**Keywords:** Theory of mind, Social cognition, Animal cognition, Developmental psychology

## Abstract

Theory of mind, the attribution of mental states to others is one form of social cognition. The aim of this paper is to highlight the importance of another, much simpler, form of social cognition, which I call vicarious representation. Vicarious representation is the attribution of other-centered properties to objects. This mental capacity is different from, and much simpler than, theory of mind as it does not imply the understanding (or representation) of the mental (or even perceptual) states of another agents. I argue that the most convincing experiments that are supposed to show that non-human primates have theory of mind in fact demonstrate that they are capable of vicarious representation. The same is true for the experiments about the theory of mind of infants under 12 months.

## 1 Theory of mind

We attribute mental states to others all the time: if I know that my wife wants diamonds for her birthday, this may influence my actions. And if I know that my daughter knows how to open her bedroom door, this may also influence my actions. We use this ability, the ability to attribute mental states – beliefs, desires, wishes, etc. – to others all the time. In fact, it would be very difficult to get by without this ability, which is known as ‘theory of mind’.

In the last paragraph, I used the term ‘theory of mind’ as a synonym for the ability to attribute mental states to others, a characterization that encompasses both the theory theory (where this attribution is the result of the application of a theory, see Nichols & Stich, 2003) and the simulation theory (where this attribution is the result of an imaginative process, see Heal, 1995, Gordon, 1995a, Gordon, 1995b). And this is indeed the original characterization given in the paper that introduced this concept: from Premack and Woodruff (1978). But it needs to be noted that there is no consensus about the exact meaning of this concept. Some talk about ‘theory of mind’ as the “capacity to reason about mental states” (Penn & Povinelli, 2007, p. 731), some others as the ability to understand others' mental states (Call & Tomasello, 2008). Peter Carruthers and Peter K. Smith in their introduction to the first edited volume on ‘theory of mind’ take it to mean possessing “a conception of the mind of another creature” (Carruthers & Smith, 1996, p. 1). Cecilia Heyes, in an influential survey article, claims that “an animal with theory of mind believes that mental states play a causal role in generalizing behavior and infers the presence of mental states in others by observing their appearance and behavior” (Heyes, 1998, p. 102). Others hold a similarly demanding view, according to which only passing the false belief test provides evidence for theory of mind (Gómez, 2004).

The differences between these formulations are significant: Heyes's concept is quite demanding: it requires the animal to represent an unobservable entity as well as a causal relation between an unobservable and an observable entity. In comparison, the original Premack and Woodruff concept is more liberal: attributing a mental state does not even require the representation of another representation (see Perner, 1991). And the recently proposed ‘minimal theory of mind’ is even more liberal (Apperly & Butterfill, 2009; Butterfill & Apperly, 2013).

There has been recently, especially in the developmental psychology literature, a move away from the use of the concept of theory of mind. But the proposed alternatives, for example, the concept of ‘perspective taking’ (Moll & Tomasello, 2006; Tversky & Hard, 2009) are still forms of attributing mental states, in this case, perceptual states to someone else. My aim is to provide a genuine alternative to the theory of mind framework in the domain of social cognition: vicarious representation.

## 2 Vicarious representation

Much of our mental life consists in representing things (objects, events, but also agents) around us. Representing something means attributing properties to this thing. And depending on what kind of properties we attribute, we get very different representational processes.

First, some of the properties we attribute to objects (or events, I will just say objects in what follows) are properties that can be fully specified without reference to any particular agent. The shape of a triangle is a property that can be fully specified without reference to any particular agent. Similarly, the color of the triangle is also a property that can be fully specified without reference to any particular agent (this is true even if we endorse theories of color according to which color is a secondary quality).

Second, we very often attribute what I call self-centered properties to objects. Self-centered properties are properties that cannot be fully specified without reference to the representing subject. They are relational properties, the two relata of which are the object and the representing subject. Egocentric spatial location (that is, spatial location indexed to our position), egocentric weight (that is, weight indexed to our lifting strength) and egocentric size (size indexed to our grip size) are self-centered properties. The attribution of self-centered properties is necessary for performing actions.

Suppose that you want to pick up a cup. In order to perform this action, you need to represent the cup as having a certain spatial location; otherwise, you would have no idea in which direction to reach. And this spatial location needs to be a self-centered one, representing the spatial location of the cup relative to where your hand is. You also need to represent it as having a certain size, otherwise you could not approach it with the appropriate grip size. Again, this size property needs to be a self-centered size property, representing the size of the cup relative to your grip size. In short, without representing objects as having self-centered properties we could not perform any bodily actions. The attribution of self-centered properties should be part of any attempt at understanding the mind.

The attribution of self-centered properties does not entail the representation of ourselves as agents. Self-centered properties are relational properties. One of the two relata is the object and the other one is the representing subject. But the representing subject does not need to be represented explicitly (as the representing subject) whenever this relational property is attributed to an object. To use the examples of self-centered properties that play a role in the performance of actions, when I reach out to grasp a cup, this does not entail representing myself as an agent. It does, however, entails representing my grip size in some way.

I defined self-centered properties as properties that cannot be fully specified without reference to the agent. But what is it about the agent that needs to be specified? Is a self-centered property indexed to the agent's spatial location? Her perspective? Her action? Her emotion? Here, we need some more distinctions.

Self-centered action-relevance properties are properties that cannot be fully specified without reference to the agent's action. If I represent something as edible for me, this amounts to the attribution of self-centered action-relevance properties. The property of being edible for me cannot be fully specified without reference to my action. The same goes for the property of being graspable for me, or, in general, for the property of affording a certain action to me. Note that the use of the term ‘affording an action’ does not imply any talk of, let alone, endorsement of, Gibsonian affordances (Gibson, 1979). Affording an action is a property we attribute to an object out there in the world – this clashes with Gibson's framework in a number of ways.^1^

Self-centered emotional relevance properties are properties that cannot be fully specified without reference to the agent's emotions. If I represent something as scary to me or disgusting to me, this amounts to the attribution of self-centered emotional relevance properties. The property of being disgusting to me cannot be fully specified without reference to my emotion (of disgust). This is not in any way a revisionary way of thinking about emotions: thinking about the representation that (partly) constitutes emotions as the attribution of self-centered emotional relevance properties is consistent with all mainstream accounts of emotion (even those accounts that do not take emotions to be a primarily representational state).

So far, I have not said anything even remotely controversial or surprising. But now enters vicarious representation. We have seen that the attributed properties can be self-centered or not centered at all. But they can also be other-centered properties, by which I mean properties that cannot be fully specified without reference to *another* agent. Other-centered properties are, just like self-centered properties, relational properties, but the two relata of this kind of relational property are the object and another subject (a subject different from the representing subject).

We need to ask, as we did in the case of self-centered properties about how the other agent specifies these other-centered properties: What is it about the other agent that the other-centered property is indexed to? Her spatial location? Her perspective? Her action? Her emotion? Again, more distinctions are needed.

Other-centered action-relevant properties are properties that cannot be fully specified without reference to the other agent's action. When I represent something as edible for someone else or as graspable for someone else (or as affording an action to someone else), this amounts to the attribution of an other-centered action-relevance property. The property of being edible for my friend Jane cannot be fully specified without reference to Jane's action (of eating).

Other-centered emotional relevance properties are properties that cannot be fully specified without reference to the other agent's. When I represent something as scary or disgusting for someone else, this amounts to the attribution of an other-centered emotional relevance property. The property of being disgusting for my friend Jane cannot be fully specified without reference to Jane's emotion (of disgust). Some of our most important social emotions work like this (Smith, 1759, Nanay, 2016, Nanay, 2018, see also Wondra & Ellsworth, 2015 for a related proposal).

Now for some terminology: vicarious representation is the attribution of other-centered properties to an object. Vicarious emotion is the attribution of other-centered emotional relevance properties to an object. One may ask: what kind of attribution is the attribution of other-centered properties to an object? In other words, what kind of representation is a vicarious representation? What is its format? Iconic or propositional? Is it a perceptual or a post-perceptual representation? I will stay neutral about these questions (Nanay, 2013, following Marc Jeannerod and some experimental work inspired by him (Jeannerod, 1994, Jeannerod & Jacob, 2005, see also Prinz, 2014, Costantini, Ambrosini, Tieri, Sinigaglia, & Committeri, 2010; Costantini, Committeri, & Sinigaglia, 2011) argued that vicarious representation is a form of perceptual representation, but I will not endorse this claim here).

Vicarious representation is a form of social cognition. And it is a less demanding form of social cognition than theory of mind, because vicarious representation does not entail the attribution of mental states to others. Vicarious representation is merely the representation of an object (as having certain special kind of properties).

It is important that both self-centered and other-centered properties are indexed to particular agents – self-centered properties to myself and other-centered properties to some other particular agent. They are not indexed to agent-types. Representing an object as something I should run away from amounts to the attribution of a self-centered property. And representing an object as something you should run away from amounts to the attribution of an other-centered property. Both of these properties are indexed to a particular subject (myself and you). They are not indexed to a type of subjects, like humans in general or living beings in general. Here is another example. Edible for me is a self-centered property. Edible for Jane is an other-centered property. Edible (which, I assume, means edible tout court or edible for humans) is neither as it is not indexed to a particular subject.

We very often represent things vicariously. When we are following a sport event on TV, say, a football game, and we cheer for team A, we represent the ball or players of team B as having various other-centered action-relevance properties (where this other is often a member of team A). When we are watching movies, we also often represent vicariously. Suppose that you are watching a Tom and Jerry cartoon, where Tom is chasing Jerry, who gets stuck in a corner where a hammer happens to be lying around. You represent the hammer as having not self-centered, but as Jerry-centered action-relevance properties – as affording a certain action not to yourself, but to Jerry (or to Tom, depending on who you're identifying with).

## 3 Vicarious representation versus theory of mind

Vicarious representation does not count as theory of mind on any of the definitions of theory of mind I mentioned in Section 1. Importantly, I can engage with someone else this way without attributing any mental (or even perceptual) state to her. Here is an example:

I am sitting in my armchair looking out of my heavily reinforced window. I see my neighbor standing in the middle of the street, deep in thought. I also see that unbeknownst to her, a tiger is approaching her from behind and it is about to attack her. I attribute other-centered (neighbor-centered) action-relevance properties to the tiger. Not a self-centered one – I am safe and sound behind my heavily reinforced window –, but other-centered ones. In this example, I know that my neighbor does not see the tiger, but I nonetheless represent the tiger vicariously: as having very salient other-centered action-relevance properties.

Note that while the tiger is an agent, nothing depends on this in the present example – if a (very quiet) train was approaching her from behind, this would not change the example. (although the tiger example is perhaps more vivid, so I will stick with it for what follows). What is crucial, however, is that my neighbor is an agent: if the tiger was about to attack a cardboard box (or if the train was approaching a cardboard box), this would not trigger vicarious representation. It might amount to representing the tiger as having cardboard box-centered properties, but cardboard-centered properties are not other-centered properties as other-centered properties are indexed to a specific agent (and a cardboard box is not an agent). We can and often do have strong emotional reaction when we see something threatening an inanimate object (that is, for some reason, dear to us). Presumably not a cardboard box, but, as in the Buster Keaton short *One Week* (1920), a portable house maybe, which, in this film, gets stuck on the train tracks with the train approaching and its owners feverishly trying to dislodge it. This example may help us to appreciate why other-centered properties are always indexed to an agent who is, at least in principle, is capable of performing some action. A cardboard box or a portable house is not capable of performing any action. When we watch the scene in *One Week*, we do not attribute portable house-centered properties to anything. We attribute Buster-centered properties (because Buster is an agent). Vicarious representation is the representation of an object (train or tiger) as having other-centered properties (that is, properties indexed to another agent).

Vicarious representation is a form of social cognition. I represent the tiger vicariously – I attribute a neighbor-centered action-relevance property to the tiger, something like the property of something my neighbor should run away from. This property is very much indexed to my neighbor: it cannot be fully specified without reference to my neighbor. But attributing this property to the tiger (or the train) does not entail attributing any mental state to my neighbor. So this instance of vicarious representation would be an example of social cognition without theory of mind.

Our cognitive (and maybe emotional) engagement with others is as strong (or maybe even stronger) if there is an epistemic asymmetry between the two agents (between me and my neighbor). If I know more than you do – if I see something close to you as a threat, whereas you don't – I am likely to react as strongly as (or maybe even more strongly than) I would if you were also aware of the threat. This very basic ‘Watch out!’ reaction is difficult to explain in terms of the attribution of mental states. What mental state am I supposed to attribute to my neighbor who is about to be attacked by the tiger? Clearly not any mental state that would involve tigers given that my neighbor is not aware of any tigers. So this way of engaging with my neighbors is not based on the attribution of mental states to her. It is based on the vicarious representation of something I perceive as a threat to her.

Defenders of the idea of theory of mind could say that I attribute the mental (perceptual?) state to you that you are *not* aware of the threat and I compare this mental state (attributed to you) with my own mental state of being aware of the threat and this comparison triggers my reaction. But this explanatory scheme presupposes extremely complex mental processes in order to explain a very simple and instinctive reaction.

In short, vicarious representation is very different from, and much simpler than, theory of mind. I am not denying that theory of mind is an important form of social cognition. But it is not the only one. And it is definitely not the most rudimentary one. I will argue in Section 5 that non-human animals and infants under 18 months are capable of vicarious representation, but not theory of mind. But in the case of adult humans, vicarious representation and theory of mind are often intertwined. And vicarious representation in adult humans is often influenced by theory of mind.

In other words, vicarious representation is a form of social cognition that comes on a spectrum. Some instances require no attribution of mental states to anyone. The tiger case would be an example. Some other instances do require attribution of mental states – in these instances vicarious representation is influenced by theory of mind. Here is an example: I go to a party with my friend Julien and the first person I see is Julien's ex, making out with another guy. I don't really care about this girl, but I know Julien still does. I will represent this event in a vicarious manner: I attribute a number of other-centered (Julien-centered) action-relevance and emotional relevance properties to this event. But my doing so very much depends on what I know about Julien. So my vicarious representation of this event is influenced by the mental states I attribute to Julien.

I said that some instances of vicarious representation require no theory of mind, while some others are influenced by it. I should make it clear, however, that all forms of vicarious representation presuppose the representation of animacy. I could not attribute other-centered properties to anything unless I represented someone as animate who these properties could be indexed to. But as the attribution of animacy does not presuppose the attribution of mental states to anyone (see, e.g., Scholl & Gao, 2013 for a summary), this is consistent with fully theory of mind-free forms of vicarious representation.

## 4 Vicarious representation versus other forms of social cognition

I have contrasted vicarious representation with theory of mind. But theory of mind is not the only account of social cognition on the market. I also need to contrast vicarious representation with two other proposals about social cognition. The first suggestion is that the most basic form of social cognition involves merely seeing the mental states (or emotions) of another person on her face (Dretske, 1973; Gallagher, 2008; Ratcliffe, 2007; Zahavi, 2008). There is no need to attribute mental states to others: the mental states of others are directly perceivable.

I am not sure how this account can be made more precise: what kind of perceptual process would count as the direct perception of someone's emotions or mental states. But whatever it is, it seems to presuppose that I see the other person's face. Note that there is no such requirement in the case of vicarious representation. If you are facing away from me, and I see a tiger attacking you from behind, we still get an instance of vicarious representation. In the case of vicarious representation, there is no need for me to see your face. All I need to see is the object that I experience as affording an action to you. The direct perception of other people's mental states (if we can make this concept more precise) may or may not be one of our ways of engaging with others. But it is very different from vicarious representation.

The third proposal about social cognition might not be a direct competitor of my view (but rather an explanation of the implementing mechanism thereof), but it is still worth highlighting the differences. It is the explanation of social cognition in terms of mirror neurons (Gallese, 2007; Gallese & Goldman, 1998; Gallese, Keysers, & Rizzolatti, 2004; Rizzolatti & Sinigaglia, 2008; Sinigaglia, 2009). The general idea is that rudimentary forms of social cognition can be explained without appealing to attribution of beliefs to others, with the help of the mirror neuron system. The mirror neuron system (or, rather, systems, as there are many mirror neuron systems in the brain, but I will focus on the one in the parieto-frontal network) consists of bi-modal neurons that get activated both when the agent performs an action and when she perceives another agent performing this action (both in rhesus monkeys and in humans: Gallese, Fadiga, Fogassi, & Rizzolatti, 1996, Umiltà et al., 2008, see Rizzolatti & Sinigaglia, 2008 for a summary). Importantly, the mirror neurons do not get activated when the perceived agent does not perform a goal-directed action (often called a ‘motor act’) but exhibits a mere (not goal-directed) bodily movement (Kakei, Hoffman, & Strick, 2001; Umiltà et al., 2008). If the other agent is grasping a ball, the mirror neurons fire, if she is making a grasping movement without there being anything to be grasped, they do not. The general proposal then is that the mirror neuron system is capable of calculating the other agent's intention from her bodily movement. In this sense, it is capable of attributing a mental state (an intention or a ‘goal-state’) to another agent.

There are some important worries about this general suggestion (not about the existence of mirror neurons but about whether they can explain our cognitive engagements with others, see Jacob, 2008, Jacob, 2009, see also Csibra, 2007). But whether or not the explanation of our cognitive engagement with others with the help of mirror neurons is a viable one, it needs to be pointed out that it is very different from my approach. The main difference is this: mirror neurons get activated only if another agent is performing an action. But vicarious representation can happen when the other agent does not do anything at all. In the tiger example above, the other agent is standing still (while the tiger, unbeknownst to her, is attacking from behind) but we are nonetheless engaging with her very strongly. The mirror neuron hypothesis, even if it is unproblematic otherwise, cannot explain this.

Vicarious representation is also different from submentalizing. Cecilia Heyes argued that many central cases of implicit social cognition can be explained as submentalizing – as the application of domain general mental processes in social context. It is important to emphasize that submentalizing does not count as genuine social cognition – it is domain-general, non-social cognition deployed in a social context: the other agent does not figure in the content of our representations at all. Importantly, the submentalizing explanations Heyes offers all amount to the attribution of either self-centered properties or non-centered properties (properties that can be fully characterized without any reference to any agent). They do not attribute other-centered properties. Hence, they are very different from vicarious representation.

After clarifying how vicarious representation is different from the most important accounts of social cognition, I need to make a couple of terminological remarks. I use the term ‘vicarious representation’ to describe the attribution of other-centered properties to objects. But the term ‘vicarious’ has also been used in other ways in the social cognition literature. Vicarious grief, for example, “refers to grief stimulated by someone else's loss” (Rando, 2002, p. 59). This is an importantly different concept of 'vicarious' from my own. In my account, what makes a representation vicarious is not that it is stimulated by a representation of another agent. As we have seen, a vicarious representation can be triggered even when we do not attribute any mental states to anyone. The same goes the concept of vicarious pain (de Vignemont & Jacob, 2012). Josef Perner uses the concept ‘vicarious file’ in his recent work, but vicarious files are not vicarious representations in the sense I am using the term – they are the representation of the other person's mental states (e.g., Perner, 2015). As we have seen, we can have vicarious representations without representing the other person's mental states.

## 5 Empirical payoff

After having clarified what form of social cognition vicarious representation is and how it differs from other forms of social cognition, I will now argue that a number of important social cognition experiments can be explained in terms of vicarious representation better than in terms of other forms of social cognition. This is true of social cognition in infants under 12 months (Section 5.1), non-human primates (Section 5.2), and also of adult humans (Section 5.3). Given that I obviously can't cover all of the many many social cognition experiments in all these three fields, I will focus on the ones that have been the most influential and/or the most difficult to explain. My general strategy is not only to offer an explanation in terms of vicarious representation for cases that allegedly necessitate the attribution of mental states, but also to explain results that, on the face of it, are difficult to explain by appealing to the attribution of mental states. So I am not only appealing to simplicity considerations in these arguments – although an explanation in terms of vicarious representation (that is, the representation of an object) is simpler than an explanation in terms of theory of mind (that is the representation of a representation of an object) on any account of simplicity (see Sober, 2015 for a detailed account of simplicity considerations in choosing between scientific theories). But vicarious representation also gives an explanatorily unified picture of social cognition. Explanatory unification is a theoretical virtue of scientific theories (Kitcher, 1981): the more diverse sets of findings a theory can explain the more unified it is. I hope to show that the concept of vicarious representation could give us a highly unified account of social cognition, bringing together infant data, non-human primate data and results about adult humans.

### 5.1 Vicarious representation and cognitive development

One big debate in the theory of mind literature concerns developmental psychology: at what age do children acquire the ability to attribute mental states to others. The initial response was age four (Wellman, Cross, & Watson, 2001; Wimmer & Perner, 1983), which quickly went down to two (O'Neill, 1996; Southgate, Senju, & Csibra, 2007), then one and a half years (Meltzoff, 1995). More recently, there are more controversial proposals for evidence concerning social cognition in fifteen month old (Onishi & Baillargeon, 2005), thirteen and a half month old (Song, Baillargeon, & Fisher, 2005), thirteen month old (Surian, Caldi, & Sperber, 2007), twelve month old (Csibra, Bíró, Koós, & Gergely, 2003; Gergely, Nadasdy, Csibra, & Biro, 1995; Kuhlmeier, Wynn, & Bloom, 2003), ten month old (Hamlin, Wynn, & Bloom, 2007), nine month old (Csibra, Gergely, Bíró, Koós, & Brockbank, 1999), and even six and a half month old (Csibra, 2008; Kamewari, Kato, Kanda, Ishiguro, & Hiraki, 2005) infants – to mention only a couple of important milestones (see also Kulke & Rakoczy, 2018 and Barone, Corradi, & Gomila, 2019 for very general overviews). None of these proposals are uncontroversial – there are suggestions for explanations of the displayed behavior without any reference to anything reminiscent of theory of mind (some examples: Perner & Ruffman, 2005, Premack & Premack, 1997).

It is important that the terminology used in these studies is not uniform. While some talk of the attribution of beliefs to others (for example, Surian et al., 2007), others talk of the attribution of goals (Csibra et al., 1999), of ‘perspective taking’ (Tversky & Hard, 2009), of the attribution of dispositions to act in a certain way (Hamlin et al., 2007; Song et al., 2005) or ‘taking the intentional stance’ (Gergely et al., 1995).

Nonetheless, in spite of all these differences, all these proposals are about the attribution of some kind of mental (maybe perceptual) state to another subject. My suggestion is that all the relevant experimental findings that are supposed to demonstrate that one year old and younger infants display the capacity to attribute mental states to others are in fact instances of vicarious representation. Let us take some of the most famous experimental findings about the development of social cognition.

(a) 13.5 month old infants who have watched an actor slide toy trucks on the floor look at an actor who grasps a toy truck that is enclosed in a small frame longer than they look at an actor who grasps an identical toy truck that is not enclosed (and is therefore free to slide) (Song et al., 2005). The authors conclude that 13.5 year olds attribute dispositions to act.
(b) One year olds who have watched a circle go around an obstacle on its way to a larger circle look at the small circle taking the detour when the obstacle is not there longer than they look at it going straight towards the larger circle without any detour (Gergely et al., 1995). The authors conclude that one year old infants “take the intentional stance” The same is true of nine-month olds (Csibra et al., 1999).
(c) Twelve month old infants prefer helpers to hinderers: if they see a triangle helping a circle up a slope, and a square trying to prevent the circle from climbing up the slope, they show preference for the triangle (Kuhlmeier et al., 2003). The authors' conclusion is that one year olds evaluate others on the basis of their social behavior and they attribute dispositions to others. The same is true of ten month old and nine month old infants, but not six month olds (Hamlin et al., 2007)
(d) The general setup of experiment (b) was replicated with six and a half year olds but the agent who the infants attributed goals was a human (not a small circle) (Kamewari et al., 2005). Later, it has been shown that the agent does not need to be a human (or even human-looking): it can be a box, as long as the route this box takes around the obstacle is varied (Csibra, 2008).

A striking feature of these experiments is that they all seem to follow the same pattern, which is in fact the pattern of vicarious representation: the infant represents an object as having other-centered action-relevance properties: as affording an action to another agent. More specifically, *the infant represents* the toy truck (a), the obstacle or lack thereof (b), (d) and the triangle or the square (c) *as affording the action* of sliding (a), of going around it (b), (d) and of helping or hindering (c) *to* the actor (a), the circle (b), (c) and the box (d).

In other words, the experimental findings (a)-(d) can be explained without any reference to the attribution of any mental state (be it belief or goal). They can be explained with the help of vicarious representation. The evidence for social cognition in infants younger than one year is in fact evidence for vicarious representation in these infants. And this evidence tells us that vicarious representation emerges sometime between month six and nine.

Take (c) as an example. The data is that the infants show preference for the triangle who helps the circle up the slope over the square who is trying to prevent the square from climbing up the slope. In the theory of mind framework, this can be explained by describing the infant as having a fairly complex mental state of attributing a disposition or maybe even virtue/vice to the triangle and the square. But vicarious representations provide a much simpler explanatory scheme: the infant does not attribute any mental state (or disposition) to anyone, she merely represents the triangle as having one other-centered (circle-centered) action-relevance property (where the action is positively valenced) and she represents the square as having another other-centered (also circle-centered) action-relevance property (where the action is negatively valenced). She represents the triangle as affording a certain action to the circle, whereas she represents the square as affording another action to the circle. On the basis of these vicarious representations, she forms an, understandable, preference for the triangle. Examples (a), (b) and (d) can be analyzed in the same way.

Some key experiments about the social cognition abilities of prelinguistic infants follow the pattern outlined above in a less straightforward manner, but I would like to argue that they nonetheless are best explained with the help of the conceptual apparatus of vicarious representation. One important example is the following experiment (Kampis, Somogyi, Itakura, & Király, 2013). 10-month old infants repeatedly see agent A choosing a yellow cube over a green pyramid many times. After this phase, the infant expects another agent, B, to choose the yellow cube over the green pyramid as well. The experimental setup cleverly rules out the obvious explanations of associative learning as well as of the infant herself preferring the yellow cube and projecting her preference to B.

The experimental setup is similar to the ones I considered above, but with a twist. During the training phase, the infant repeatedly represents the yellow cube as having one other-centered (that is, A-centered) action-relevance property and represents the green pyramid as having another other-centered (that is, A-centered) action-relevance property. And in the trial phase she represents the yellow cube as having a B-centered action-relevance property that is similar to the A-centered action-relevance property she has previously represented the yellow cube as having. Ditto for the green pyramid: she represents the green pyramid as having a B-centered action-relevance property that is similar to the A-centered action-relevance property she has previously represented the green pyramid as having.

To put it more informal terms, she has represented the yellow cube as affording a certain action to A and now when B is around, she represents the yellow cube as affording the same action to B. The authors of this study take this experiment to show that the infant binds A's preference for the yellow cube to the yellow cube and not to A. That is, to the object, not to the agent. The infant learns something about the object and not (just) about the agent. And this is consistent with my interpretation. In the training phase, the infant attributes one property to the object (that is indexed to A) and in the trial phase she attributes a similar property to the same object (albeit one that is now indexed to B).

We can describe this scenario without presupposing any theory of mind (entailing the attribution of preferences to the new agent, B) in 10-month old infants, with reference to vicarious representation only. But this experiment highlights the importance of clarifying just how thickly or thinly the other subject needs to be specified in the case of vicarious representation. The infant could shift seamlessly from the attribution of A-centered properties (in the training phase) to the attribution of B-centered properties (in the trial phase) because A and B are specified very thinly – so thinly that the attribution of A-centered properties to the yellow cube serves as a very good guide for the attribution of B-centered properties to the yellow cube.

And in many important experiments about social cognition in infants under 12 months the other agent is even more thinly specified. As we have seen, vicarious representation comes on a spectrum. When I represent something as having other-centered properties, this ‘other’ can be more or less fully specified. In the example I gave in Section 3, where I go to a party with my friend, Julien and see my friend's ex and represent her as having Julien-centered properties, this is an instance of vicarious representation where the ‘other’ needs to be quite fully specified. In order for me to have this vicarious representation, I need to know a lot about Julien's mental life. This is vicarious representation heavily intertwined with and influenced by theory of mind. This is one end of the spectrum.

On the other end of the spectrum, we have instances of vicarious representation where theory of mind plays no role whatsoever. When we attribute other-centered properties to an object, we do not need to know anything about the mental life of this ‘other’. It is enough if we know that it is a subject who occupies a certain point of view. The Kampis et al. (2013) experiment is a good example: the infant could shift from the attribution of A-centered properties to the attribution of B-centered properties because the difference between A and B, is, from the point of view of vicarious representation, negligible. They are different agents, but they are agents who occupy the same point of view.

Another famous experiment that could be explained as an example of vicarious representation where the other subject is very thinly specified in Kovács, Téglás, and Endress (2010)'s experiment on 7-month old infants (I will focus on Experiment 5 about the contrast between the P-A- and the P-A+ conditions (ball moves with the onlooker present vs. ball moves with the onlooker absent)). The way these infants represented the objects on the screen depended on whether there was a completely passive onlooker in the scene. This passive onlooker (a Smurf figure) did nothing and was completely external to what happened to the objects. Nonetheless, the mere presence of this passive onlooker automatically changed the way the infant represented the objects (the effect went away if the passive onlooker agent was replaced by an inanimate object).

The authors of the Kovács et al. (2010) paper framed this as the infant automatically keeping track of the Smurf's beliefs. The infant looks longer at the absent object when the Smurf ‘should’ believe that the object is present because the infant computes the Smurf's beliefs about the presence/absence of the object as well as her own. And if there is a discrepancy, this increases looking time.

Here is a much simpler explanation in terms of vicarious representations (and one that is not susceptible to the objections raised against the Kovacs et al. framework in Phillips et al., 2015): The infant automatically attributes Smurf-centered properties to the object whenever the Smurf is present. This does not entail attributing beliefs to the Smurf. And the mere automatic attribution of Smurf-centered properties to the object would increase the infant's looking time in P-A+ (as the Smurf-centered properties the infant attributes to the object are very different from the self-centered ones she attributes to the same object), but it would not increase the infant's looking time in P-A- (as the Smurf-centered properties the infant attributes to the object are the same as the self-centered ones she attributes to the same object). Crucially, the Smurf-centered properties the infant attributes to the object only specify the Smurf very thinly – only in terms of the Smurf's point of view. So the Kovacs et al. experiment is an example of vicarious representation, but it is an example of vicarious representation where the other agent is specified very thinly.^5^ The same is true of the experiment reported in Kampis, Parise, Csibra, and Kovacs (2015), where 8-month old infants automatically encode objects that are occluded from someone else's perspective (showing the same gamma signature as for objects occluded from their own perspective).

To sum up, the experiments for early social cognition in developmental psychology say little about the attribution of mental states. They do, however, give us a firm understanding of the emergence of vicarious representation in infancy. The evidence these experiments suggest is that vicarious representation emerges shortly after the infant turns six months old.

### 5.2 Vicarious representation in nonhuman primates

It has been severely debated in primatology and cognitive science in general whether nonhuman primates have a ‘theory of mind’, that is, whether they are capable of attributing mental states to other agents (Premack and Woodruff, 1978, Heyes, 1998, Gómez, 2004, Tomasello & Call, 1997, Tomasello, Call, & Hare, 2003, Povinelli and Vonk, 2003). There seems to be consensus on only one claim in this literature, namely, that on some interpretations of what theory of mind is, chimpanzees do have theory of mind, whereas on others, they don't (see Call & Tomasello, 2008 for a summary).

The best candidate for primate theory of mind that most recent research is about is ‘perspective taking’: it has been argued that chimpanzees respond differentially to what they take the other agent to see (Call & Tomasello, 2005; Hare, Call, Agnetta, & Tomasello, 2000; Hare et al., 2001, Hare et al., 2006; Tomasello et al., 2003; Tomasello & Call, 2006). The key experiment here is one where a dominant and a subordinate chimpanzee spontaneously compete for food. One food item is visible for both of them, whereas the other one is only visible for the subordinate chimpanzee. It turns out that the subordinate vastly prefers to go for the one that the dominant does not see. Although the results of this experiment have been disputed (see esp. Karin-D'Arcy & Povinelli, 2002), they have been successfully replicated with sufficient control (Bräuer, Call, & Tomasello, 2007).

A straightforward way of explaining this behavior would be to say that the subordinate takes into consideration what the dominant sees. In short, it attributes a perceptual state to the dominant chimpanzee.

Should we then conclude that chimpanzees are capable of attributing perceptual states to others? The problem is that some other experimental findings contradict this claim. Daniel Povinelli and his coworkers conducted a series of experiments that seem to demonstrate that chimpanzees do not attribute perceptual states to each other (Povinelli & Vonk, 2003, Povinelli & Eddy, 1996, Penn & Povinelli, 2007, Reaux, Theall, & Povinelli, 1999, see also Bulloch, Boysen, & Furlong, 2008). The most decisive set of experiments is the following: the chimpanzee can ask for food from two agents, only one of whom seems to be able see (say, because the other one has a bucket on her head). It turns out that chimpanzees ask for food from the two agents, regardless of which agent seems to be a perceiver. These experiments seem to show that chimpanzees do not attribute even perceptual states to others. If they did, they would show a preference for asking for food from those agents who seem to be able to see them. But they do not show any such preference. It seems then that even the most plausible candidate for theory of mind, namely, the attribution of perceptual states to others, lacks conclusive support (see also Sterelny, 2003).

And here is the point where the concept of vicarious representation can be applied successfully. The Hare et al. experiments can be appropriately described as instances of vicarious representation. The subordinate attributes some dominant-centered action-relevance properties to one food item and some other dominant-centered action-relevance properties to the other food item and then compares these two vicarious representations. In other words, the subordinate represents the food item as affording the action of eating to the dominant and she represents the other food item as not affording the action of eating to the dominant. And she, understandably, goes for the one that does not afford eating to the dominant. This process can be described entirely in terms of vicarious representation, and vicarious representation that specified the other agent only very thinly (only specifying the agent's point of view).

Note that all the supposed positive experiments in favor of the existence of theory of mind in non-human primates work on the very same model, starting with Premack and Woodruff's original ones: the chimp in that experiment represents objects (the key, the tap, the switch, the box) as having other-centered (experimenter-centered) action-properties (involving actions like opening the cage, rinsing the dirty floor, lighting the unlighted heater, reaching the bananas). In other words, the chimp represents these objects vicariously, as affording various actions to the experimenter.

And the same is true of the alleged anecdotal evidence for theory of mind in non-human primates. One of the earliest such anecdote is the following (Goodall, 1971): a young chimpanzee noticed a piece of food on a tree, but did not get it and did not even look at it as long as there were other chimps around, but when they left, she immediately jumped up and grabbed it (see also Byrne & Withen, 1988 and de Waal, 1982 for similar anecdotes). Again, the young chimp represented the food item as affording the action of eating to the dominant chimpanzee and acted on this vicarious representation.

The Povinelli experiments are somewhat more difficult to handle. An important contrast here is the following set of experiments with rhesus monkeys (Flombaum & Santos, 2005).^6^ The experimenters looked exactly the way they looked in the Povinelli experiments: one blindfolded, the other not blindfolded, for example. They even stood in a way very similar to the experimental setup in the Povinelli scenarios. The big difference was that the monkeys were in a competitive situation: the monkeys wanted to snatch the food from the experimenter – instead of asking him to give it to them. And they did indeed tend to steal the food only from the blindfolded experimenter and not the other one.

Note that the Flombaum and Santos experiments fit the vicarious representation pattern perfectly: the monkey represents the food as affording an action (of taking it) for one of the experimenters, but not to the other. And as the monkey is afraid of the experimenter, she will only go for the food if it does not afford this action (the action of taking the food, thereby preventing the monkey from taking it) to the experimenter. So it attributes two very different experimenter-centered action-properties to the food item (indexed to the two experimenters). This is a structurally very similar situation to the one in the Hare et al. experiments. The fact that the way the experimenters look is almost identical to the Povinelli experiments, however, offers us a neat way of comparing the alleged pro and the alleged con cases of primate theory of mind.

First, we have no reason to assume that the chimp in the Povinelli experiments represents the food in a vicarious manner. We have no reason to suppose that the chimp attributes experimenter-centered action-relevance properties to the food. Povinelli's own explanation amounts to saying that the chimp represents the food as having self-centered action-properties – she represents the food as affording an action to herself and the experimenter merely figures in this action as a means of achieving its goal. We can fully explain the chimp's behavior without talking about vicarious representation (a claim Povinelli would wholeheartedly agree with). In the Hare et al. and the Flombaum and Santos experiments, in contrast, we need to posit vicarious representations. In order to explain why the chimp responds differentially to the two experimenters, we need to assume that the chimp represents the food as having two different experimenter-centered action-relevance properties (different depending on the experimenter in question). In short, there is vicarious representation at work in the Flombaum and Santos experiments, but not in the Povinelli experiments.

The emphasis on vicarious representations can also help us to understand why apes and monkeys tend to do much better at competitive mind-reading tasks than in collaborative ones. The Hare et al. experiment is a competitive situation: the subordinate chimp is competing with the dominant one for food. The anecdotal evidence described in Goodall, 1971 is also a competitive one and so is the Flombaum and Santos experiment. A number of recent findings reported in Kano, Krupenye, Hirata, Tomonaga, and Call (2019), Krupenye, Kano, Hirata, Call, and Tomasello (2016), and Buttelmann, Buttelmann, Carpenter, Call, and Tomasello (2017) also all count as competitive. The Povinelli experimental setup, in contrast, is a collaborative situation. One may be tempted to explain the difference between the results as a consequence of the difference in terms of the competitiveness/collaborativeness of the situation.

While this contrast has been made repeatedly in the literature (see Tomasello, Carpenter, Behne, & Moll, 2005 for a summary), it is important to note that appealing to the difference between competitive and cooperative situations only shifts the explanatory burden. If chimps do not attribute perceptual states in the Povinelli experiments because it is a cooperative situation and they do so in the Hare et al. experiments because it is a competitive situation, this just raises an even more fundamental question: why should the competitive/cooperative distinction make a difference in the attribution of perceptual states. Further, if chimps do have the capacity to attribute perceptual states (as evidenced by the competitive situations), what stops them from using this capacity in cooperative situations if it benefits them?

One major advantage of the vicarious representation framework is that it may help us to elucidate this distinction. The big difference between the Povinelli setup and the Hare et al. setup then is the following. In both of these scenarios, there are two potential actions: the food is represented as affording one potential action to the dominant chimp/the experimenter and it is represented as affording another potential action to the (subordinate) chimp. In the Hare et al. scenario, these two actions are incompatible: it is either the dominant or the subordinate chimpanzee who gets to eat the food. In the Povinelli scenario, the actions are not incompatible: in fact, the experimenter's action is a way of performing the chimp's action. That is why there is no vicarious representation in the Povinelli scenario, while there is vicarious representation in the Hare et al. scenario.

Here is another way of putting it. The chimpanzee (or the subordinate chimp) represents the food as having self-centered action-relevance properties and as having other-centered (that is, experimenter/dominant chimp-centered) action-relevance properties. The question is the relation between the two actions that specify the self-centered and the other-centered action-relevance properties. In competitive scenarios, the performance of one of these excludes the performance of the other (like in the Hare et al. experiments). In cooperative scenarios, in contrast, the performance of the two actions is compatible – performing one is a means of performing the other (like in the Povinelli experiment).

To generalize from these two cases, in competitive scenarios, the action the other agent (is about to) perform is incompatible with the action the subject (is about to) perform. In cooperative scenarios, there is no such incompatibility. Hence, in competitive situations, the subject needs to consider what action objects afford to the other agent – she can't just consider her own action. This amounts to having vicarious representation, which non-human primates are capable of. In the cooperative situation, in contrast, the subject needs to do something very different and much more complicated: she would need to consider not just the other-centered properties of an object, but also the other-centered properties of an action that the object affords. In the Povinelli setup, the chimp would need to consider what action is afforded to the experimenter by the action of her own begging gesture. In short, she would need to keep track of one action (by the other agent) afforded by another action (by she herself). And this would amount to having some kind of second-order action-representation, which is a much more complex mental state than vicarious representation. The real difference between competitive and cooperative situations is about actions – more precisely about the compatibility/incompatibility of the actions afforded to the subject and to the other agent. The theoretical framework of vicarious representation can explain this difference. The theory of mind framework cannot.

I argued that we can explain the seemingly conflicting results of the Hare et al. and the Povinelli experiments as instances of vicarious representation. Further, it seems that chimpanzees are capable of this (and maybe only this) way of engaging with other agents. Some of the controversy around the empirical findings about chimpanzee theory of mind could therefore be resolved in the sense that there seems to be solid evidence that primates are capable of vicarious representation, even if one is skeptical of the evidence that they have the ability to attribute mental states to each other. Making a distinction between vicarious representation and theory of mind can help us understand the social cognition skills of non-human primates better.

### 5.3 Vicarious representation in adult humans

The third big debate about the concept of theory of mind is not about preverbal infants or non-human primates, but about adult human beings. We attribute beliefs and desires to each other all the time. The question is: how do we do it? What are the mental processes that make the cognitive engagement of adult humans with others possible?

We have seen that in adult humans vicarious representation and theory of mind are often intertwined. But this does not mean that we can't find empirical evidence for the importance of vicarious representation in adult humans.

One important finding is that we (adult humans) are much more likely to spontaneously describe things from someone else's perspective when the performance of an action is involved in some way (Tversky & Hard, 2009). If we stick to the theory of mind framework, this result seems puzzling: whether the scenarios involve actions (in various ways) should not, on the face of it, make a difference to the attribution of mental states to the other agent, so this could not explain why the subject tends to describe the scenario from this other agent's spatial perspective. If we accept, however, that our most basic way of engaging with others cognitively is vicarious representation, then these results are exactly what we should expect: we shift perspective when we represent objects around us as affording actions to others.

Another experiment that is very relevant here is the following (Ward, Ganis, & Bach, 2019). Just as in the case of the Kovacs et al. experiment I talked about in Section 5.1, a completely irrelevant agent's presence alters performance on a task that is completely independent from the agent's presence. In this case, the performance of the mental rotation task depends on whether there is a completely passive agent in the scene. The agent does not do anything, but her presence influences the reaction time on the mental rotation task. The effect goes away if the agent is replaced by an inanimate object. As the Kovacs et al. experiment, this is also an example of vicarious representation where the other agent is very thinly specified – all that needs to be represented about her is her spatial point of view. Vicarious representation of this kind does not presuppose any attribution of any mental states to this agent.

One important empirical debate about the mental processes that make adult human social cognition possible concerns the Social Simon Effect. In the standard Simon task, the participant carries out a spatially defined response to a non-spatially defined stimulus, but the location of this (non-spatially defined) stimulus influences the response time: responses are faster if stimulus location and response location correspond. For example, if the subject sees a triangle, she is supposed to push a button on her right and if she sees a square, she has to push a button on her left. When the triangle appears in the right hand side of her visual field (or if it appears together with some marker that emphasizes that side of her visual field), her reaction is faster than it is when it appears on the left. This is the standard Simon Effect (Simon, 1990). The Social Simon Effect replicates this result in a scenario where the two different responses are carried out by two different people: if there is another agent on my left pushing a button when seeing a triangle, my reaction to the square is faster when it appears on the right than it is when it appears on the left. This difference disappears if there is no-one on my left.

The original interpretation of the Social Simon Effect was that it demonstrates that we have ‘action co-representations’ when we perform joint actions with others: a representation of both one's own action and the other person's action (Sebanz, Bekkering, & Knoblich, 2006; Sebanz, Knoblich, & Prinz, 2003): “. . .if co-actors represent each other's actions, an irrelevant spatial cue referring to the other's action should activate the representation of the other's action and create a response conflict” (Sebanz, Knoblich, and Prinz, 2005, p. 1234). It is easy to see that having an ‘action co-representation’ entails attributing a mental state to the other agent: it entails ‘theory of mind’.

But this is not the only interpretation. It has also been suggested that the reason for the Social Simon Effect is that the other agent provides a spatial reference frame – the other person's mind does not play any role in creating the effect: she is relevant only for helping us to localize the stimulus and the response in space (Dolk et al., 2011; Guagnanoa, Ruscoli, & Umilta, 2010). On this interpretation, the Social Simon Effect is not social at all – it does not involve any form of social cognition.

Neither of these interpretations is unproblematic. The problem with the first, ‘action co-representation’ interpretation is that the Social Simon Effect is also present when the subject is a subject with autism spectrum disorder (Sebanz, Knoblich, Stumpf, & Prinz, 2005). But it is widely held that at least some forms of autism can at least partly be explained in terms of the subjects' deficiency of ‘theory of mind’ (Senju, Southgate, White, & Frith, 2009). But then how is it possible that they are capable of forming ‘action co-representations’ (see also Humphreys & Bedford, 2011)? Also, it turns out that the further away the agents sit from each other, the weaker the effect gets (Guagnanoa et al., 2010). It is not at all clear why this would make a difference if the effect is to be explained by a version of ‘theory of mind’.

The problem with the second, ‘spatial reference frame’ interpretation is that the Social Simon Effect depends on the actor's bad mood (Kuhbandner, Pekrun, & Maier, 2010), and, importantly, her negative relationship to the other actor (Hommel, Colzato, & Van den Wildenberg, 2009). Further, if the agent believes that the co-actor is a computer, the effect disappears (Tsai, Kuo, Hung, & Tzeng, 2008). These findings indicate that there must be something ‘social’ in the Social Simon Effect.

The concept of vicarious representation can help us to resolve this debate. The Social Simon Effect can be interpreted as a manifestation not of ‘theory of mind’, but of vicarious representation. The effect is present because the actor is aware of the action the stimulus on the left hand side affords to her co-actor. The actor represents the stimulus on the left hand side as affording an action not to herself but to her co-actor. The actor attributes other-centered, that is, co-actor-centered action-properties to the stimulus.

This does not entail attributing any mental states to the co-actor – which explains why the effect is still present in the case of autism spectrum disorder subjects. But it does involve social cognition, namely, vicarious representation – which explains why the effect is sensitive to the agent's mood, to the relationship between the agents and to whether the agent thinks that the co-actor is a computer. As in the case of non-human primates and preverbal infants, explaining Social Simon Effect by appealing to vicarious representation is a novel, third way between explaining the effect with the help of (some version of) ‘theory of mind’ and explaining it as not involving any social cognition whatsoever.

## 6 Self first or other first?

We have seen that an object can be represented in self-centered, other-centered and non-centered manner. Representing it in an other-centered manner is what constitutes vicarious representation. Given that I argued that this form of other-centered cognition happens at a very early age, this raises a very big-picture question about what comes first: the representation of the self or the representation of the other.

The question goes back at least as far as Piaget's work, who argued that the representation of others is based on, and comes after, the representation of the self (Piaget, 1928). We represent ourselves and this gives rise to our representation of the other. This is true ontogenetically as well: infants are capable of representing themselves before they become capable of representing others. And this has become the mainstream view in developmental psychology about the relation between representing the self and representing the other.

This consensus has been broken recently. In a series of papers, Victoria Southgate argues persuasively that it's the other way round: the representation of the other comes first and it is the representation of the other that the representation of the self is based on (see esp. Southgate, 2020). This move can help us to explain why there is no conflict between other-representation and self-representation in early infancy: because the self-representation is not there.

I want to propose a third possible way of relating self-representation and other-representation. First, the terms ‘the representation of the self’ and ‘the representation of the other’ cover a lot of different kinds of representations. But we can narrow down the question about the attribution of self-centered and of other-centered properties. The question remains: what comes first?

The Piagetian answer would be that infants start out representing objects as in a self-centered manner and then this gives rise to other-centered representations. The Southgate-ian answer would be the exact opposite. I want to propose a form of compromise between these two approaches. Self-centered and other-centered representations mutually strengthen and reinforce one another. We have fairly clear evidence that infants start executing goal-directed actions at around 4 month and we have seen that this ability presupposes the attribution of self-centered action-relevance properties to the object they are manipulating. We have also seen that a couple of months later infants are capable of attributing other-centered action-relevance properties. And this, in turns makes it possible for the infant to attribute more complex and more highly specified self-centered properties, which then leads to the attribution of even more complex and more highly specified other-centered properties, and so on.

I will not defend this view here, nor will I examine the same question in the context of not the ontogeny but of the phylogeny of self-representation versus other-representation. Nor will I address where non-centered representations come in (see Nanay, 2013). But one advantage of taking vicarious representation seriously is that it opens up thus far not viable theoretical possibilities in this debate.

## 7 Conclusion

I argued that we should shift the emphasis in the most important contemporary debates about social cognition from theory of mind to vicarious representation. Theory of mind is an important form of social cognition. We have known that for 40 years. My aim was to argue that vicarious representation is also an important form of social cognition, which could help us make progress in debates concerning social cognition in non-human primates, infants under 12 months and even adult humans.

## CRediT authorship contribution statement

This is a single authored paper.

## Footnotes

1 Gibson was an anti-representationalist. I am not. Gibson also took affordances to be (half-normative) entities out there in the world. I assume, with the rest of the history of psychology, that there are no half-normative entities out there in the world, only objects. We can represent these objects in various ways. And we can also represent them as affording various actions.

5 I should add that if the other agent is specified very thinly, then the action the other-properties action-relevance properties will also specify the action in question very thinly.

6 The Flombaum and Santos experiment is about rhesus monkeys and not chimpanzees, but as rhesus monkeys are widely held to be less capable of theory of mind than chimpanzees (see Cheeny & Seyfarth, 1990), this asymmetry can be ignored.
